# Wearable technology and live video conferencing: The development of an affordable virtual teaching platform to enhance clinical skills education during the COVID-19 pandemic

**DOI:** 10.36834/cmej.70554

**Published:** 2020-09-23

**Authors:** Lauren Wintraub, Mary Xie, Mariam Issa, Yaanu Jeyakumar, Matthew Nelms, Deepanshu Sharma, Daniel Teitelbaum, Mirek Otremba, Giovanna Sirianni, Joyce Nyhof-Young, Fok-Han Leung

**Affiliations:** 1 Faculty of Medicine, University of Toronto, Ontario, Canada; 2 Division of General Internal Medicine, Mount Sinai Hospital, Ontario, Canada; 3 Department of Family and Community Medicine, University of Toronto, Ontario, Canada; 4 Sunnybrook Health Sciences Centre, Ontario, Canada; 5 Office of Assessment and Evaluation, Faculty of Medicine, University of Toronto, Ontario, Canada; 6 Department of Family and Community Medicine, St. Michael’s Hospital, Ontario, Canada

## Introduction

The novel coronavirus disease 2019 (COVID-19) pandemic has forced physical distancing, compelling most medical programs to move their entire pre-clerkship curricula online. Unfortunately, clinical skills education programs are experiencing serious challenges in this setting. Specifically, all pre-clerkship medical students at the University of Toronto have been barred from entering clinical environments for educational purposes since March 2020 and for the foreseeable future. As a result, the University of Toronto’s medical school has pivoted to teaching clinical skills using online modules and pre-recorded videos of physicians taking patient histories and performing physical examinations. Medical students and teachers have become concerned about the resulting gaps in clinical education. Their concerns stem from students’ lack of active engagement with patients and tutors, which is paramount for developing bedside manner and clinical competencies. As the pandemic persists, students continue to voice their growing concern over this pause in their face-to-face clinical skills education and the effect this will have on their learning and subsequent performance in clerkship.

Given these new obstacles, a group of pre-clerkship medical students and faculty at the University of Toronto teamed up to investigate this issue with the users in mind. Wearable technologies can provide students with rich, interactive, and resource-efficient field experiences in the absence of in-person clinical training.^1^^,^^2^ However, few studies have evaluated the use of point-of-view filming with wearable devices for medical education.^3^ Combined with live video conferencing (VC), these technologies can simulate the interactive learning of an in-person classroom while allowing students to maintain physical distancing requirements.^1^ Tutor use of wearable livestreaming devices can allow students to regain many of the benefits of the regular clinical skills curriculum.

In this commentary, we identify an affordable and user-friendly device-accessory pairing compatible with live VC technology that can be adapted for use throughout medical or other healthcare education programs.

## Potential solutions

With the support of faculty, our medical student group first conducted an online environmental scan of useful technology available for online purchase and delivery. Ultimately, we chose and reviewed seven digital device-accessory pairs (See Appendix A).

Each device-accessory pair was assessed while performing either a precordial or abdominal exam. These exams are central to undergraduate clinical skills education and have notable shortcomings in traditional pre-recorded learning aids (e.g. poor visualization of physical exam maneuvers).

The medical student team reviewed the recordings, discussed findings, and reached consensus. We then assigned each device-accessory pair a maximum of 10 points according to a scoring system based on the following key criteria:

1. Visualization of physical exam maneuvers
  - Versatility – Visualizes multiple different body parts and angles for different exams
  - Pedagogical quality – Provides views highlighting anatomy, pathology, and maneuvers
2. Ease of use
  - Setup – Minimal effort is needed to don and/or assemble equipment
  - Minimal interference – Easy to don and sanitize given COVID-19 personal protective equipment (PPE) precautions, comfortable, unobtrusive, requires minimal adjustment during exams
3. Live VC abilities
  - VC capability – Connects directly to VC technology (e.g. Zoom^©^ software)
  - VC compatibility – Requires intermediary software to connect to VC technology
4. Footage Quality
  - Video quality – Visualizes anatomy, abnormal findings, etc.
  - Audio quality – Clearly captures percussions, voices, etc.
  - Camera stability – Remains steady while in use (minimal shaking)
5. Cost
  - Affordability – Under $200 CAD (assuming physician tutor has smartphone or tablet)

We assessed and scored the seven device-accessory pairs (Table 1).

**Table 1:** Criteria fulfillment and scoring of device-accessory pairs in physical exam demonstrations.

	Versatility of view	Pedagogical quality of view(s)	Ease to setup	Minimal interference	Direct VC capability	VC capability	Video quality	Audio quality	Camera stability	affordability	Total score
Gopro^©^+Chest starp	✓	✓		✓		✓	✓	✓	✓		7
Gopro^©^+wrist strap	✓	✓				✓	✓	✓			5
Gopro^©^+patient head strap						✓			✓		2
Gopro^©^+head strap		✓			✓	✓	✓		✓	✓	6
Smartphone+ GorillaPod^©^	✓	✓			✓	✓	✓		✓	✓	7
Tablet+tripod			✓		✓	✓			✓	✓	5
Smartphone+chest strap	✓	✓	✓	✓	✓	✓	✓	✓	✓	✓	10

VC = video conferencing.

All tested modalities provided clear recordings with high quality footage. Notably, the modalities using GoPro *^©^* devices and body-mounted smartphones provided excellent visualization of physical exam anatomy and maneuvers. However, all wrist and head straps were incompatible with COVID-19 PPE requirements. Furthermore, the GorillaPod, *^©^* head straps, wrist strap, and tablet mount were cumbersome to don, set up, adjust mid-exam and produced less pedagogical views. The patient-mounted GoPro^©^ and head strap did not capture footage beneficial for learning. All three GoPro *^©^* modalities were less affordable than all other device-accessory pairs, given that physician educators do not commonly own them. Their lack of direct VC capabilities also increases concerns about security and cost.

The chest-mounted smartphone holder was affordable ($65 CAD), easy to don and use, unobtrusive, and produced high quality audio and video content clearly highlighting relevant anatomy and maneuvers. The chest mount featured adjustable elastic straps that fit comfortably on both male and female users. This modality also connected directly with video conferencing technology. The chest brace did not present sanitation/infection control challenges as it could easily be wiped down or sprayed with disinfectant.

After testing seven device-accessory pairings, we determined that a chest-mounted smartphone was the best device-accessory pair for physical exam demonstrations (Table 1). The chest mount is especially useful as it fits any smartphone, rotates easily between portrait and landscape modes, and can provide a magnified field of view to capture examination details. The chest mount harness was also made of material that could be easily wiped down or sprayed with disinfectant between uses. A different research group demonstrated the feasibility of affixing an iPad with Zoom^©^ capability to a computer on wheels to teach bedside clinical skills with COVID-19 positive patients.^4^ Two simultaneous views on a Zoom^©^ video call easily transforms our platform into a multi-perspective learning tool.

Zoom^©^ video conferencing software was also successfully used in this study and was deemed secure by hospitals associated with our medical program. Tutors who already own smartphones can easily couple them with institution-provided chest straps and Zoom^©^ accounts to resume group teaching of clinical skills. Furthermore, using this platform with Zoom^©^ technology can allow individual tutors to communicate with a far greater number of students. Through video conferencing sessions from a physician’s point of view, students will regain a crucial part of their pre-clerkship clinical skills training: the ability to interact with and ask questions of tutors and volunteer or standardized patients in real time. The affordability, versatility, and sanitation-friendly nature of our novel platform makes it ideal for implementation during the pandemic and bridges the gap in current virtual teaching affecting educational healthcare institutions globally.

Start-up costs for this intervention can be adjusted based on the number of students per learning group. Testing will be important to determine the maximum number of students one clinical skills tutor can effectively teach using this platform.

## Conclusion

We have demonstrated that the use of a chest-mounted smartphone with Zoom^©^ VC technology is an interactive, accessible, cost-effective, and feasible adaptation for virtual clinical skills teaching in real time. This platform can help address key limitations of current online curricula by creating a synchronous learning environment that simultaneously allows student-tutor discussion and clear visualization of clinical interactions. We hope that our innovation, coupled with our ongoing studies of this platform, will help transform distanced clinical skills education by providing educational institutions with tools to bridge the gap created by the pandemic.

## References

[1] Sultan N Reflective thoughts on the potential and challenges of wearable technology for healthcare provision and medical education. Int J Inf. 2015;35(5):521-6 10.1016/j.ijinfomgt.2015.04.010

[2] Thomson FC, Morrison I, Watson WA ‘Going Professional’: using point-of-view filming to facilitate preparation for practice in final year medical students. BMJ Simu Technol Enhanc Learn. 2018;4(3):148-9. 10.1136/bmjstel2017-000224 PMC8990222 35520467

[3] Jeyakumar Y, Sharma D, Sirianni G, Nyhof-Young J, Otremba M, Leung FH Limitations in virtual clinical skills education for medical students during COVID-19. Can Med Educ J. 2020 May. 10.36834/cmej.70240 PMC7749689 33349770

[4] Hofmann H, Harding C, Youm J, Wiechmann W Virtual Bedside Teaching Rounds on Patients With COVID-19. Med Educ. 2020 10.1111/medu.14223 PMC7273015 32403185

